# Perceived changes in the mental well‐being among Nigerians due to Ramadan Intermittent Fasting during the COVID‐19 pandemic

**DOI:** 10.1002/brb3.2990

**Published:** 2023-04-14

**Authors:** Sahabi Kabir Sulaiman, Fatimah Isma'il Tsiga‐Ahmed, Teresa Arora, MoezAlIslam E. Faris, Muhammad Sale Musa, Yesir Adeyemi Kareem, Farouq Muhammad Dayyab, Aminu Hussein, Shehu Sale, Syed Fahad Javaid, Moien AB Khan

**Affiliations:** ^1^ Department of Medicine Yobe State University Teaching Hospital Damaturu Nigeria; ^2^ Department of Community Medicine Bayero University Kano/Aminu Kano Teaching Hospital Kano Nigeria; ^3^ College of Natural & Health Sciences Zayed University Dubai United Arab Emirates; ^4^ Department of Clinical Nutrition and Dietetics, College of Health Sciences University of Sharjah Sharjah United Arab Emirates; ^5^ Department of Medicine Yobe State University Teaching Hospital Damaturu Nigeria; ^6^ Department of General and Geriatric Psychiatry Federal Neuropsychiatric Hospital, Maiduguri Nigeria; ^7^ HIV and Tuberculosis Unit Infectious Diseases Hospital Kano Nigeria; ^8^ Department of Family Medicine Yobe State University Teaching Hospital Damaturu Nigeria; ^9^ Department of Child Psychiatry Federal Neuropsychiatric Hospital, Kware Sokoto Nigeria; ^10^ Department of Psychiatry Bayero University Kano Kano Nigeria; ^11^ Health and Wellness Research Group, Department of Psychiatry and Behavioral Sciences, College of Medicine and Health Sciences United Arab Emirates University Al‐Ain United Arab Emirates; ^12^ Health and Wellness Research Group, Department of Family Medicine, College of Medicine and Health Sciences United Arab Emirates University Al‐Ain United Arab Emirates; ^13^ Primary Care NHS North West London London United Kingdom

**Keywords:** feeding behavior, mental health, Muslims, psychology and religion, religion

## Abstract

**Introduction:**

Muslims fast every year during the month of Ramadan from dawn until dusk. This study examined mental well‐being and correlating factors among Nigerian adults who observed Ramadan intermittent fasting (RIF).

**Methods:**

A validated generalized anxiety disorder‐2 and Patient Health Questionnaire‐2, the four‐item spiritual well‐being index, and the Islamic intrinsic religiosity questionnaire were used to collect data about mental well‐being (depression, anxiety), spirituality, and intrinsic religiosity through a web‐based survey between the May 9, 2021 (27th of Ramadan, 1442) and the June 4, 2021 (29th of Shawwal, 1442). We studied the mental well‐being of respondents over a period of 4 weeks before Ramadan (BR) and during the 4 weeks of Ramadan between the April 12, 2021 and the May 12, 2021(DR). Multinomial regression analysis was used to determine the factors associated with depression and anxiety. This research did not receive any grant or funding.

**Results:**

A total of 770 adult Nigerians who observed RIF study were included in this cross‐sectional study. When compared to mental well‐being BR, observing RIF by Nigerian adult respondents was associated with a significant improvement in their mental well‐being. A higher proportion of respondents felt less depressed DR (61.3% vs. 56.5%. < .001). Interest and pleasure in doing things improved DR than BR (*p*= 0.007) and respondents felt less nervous and anxious (60.7% vs. 57.1%, respectively; *p* <.001). Mental well‐being was independently associated with sociodemographic characteristics, physical activity, and perceived relationships.

**Conclusions:**

This study found significant improvement in mental well‐being DR compared to BR despite the ongoing COVID‐19 pandemic. The effect of RIF on mental well‐being needs further research with multicentric studies among different sets of ethnic populations.

## Graphical abstract

Ramadan Intermittent fasting is associated with improved mental well‐being. Further multi‐centric research is required to guide the utilization of fasting for the benefit of society.

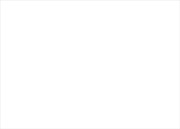

## 1 INTRODUCTION

During the annual fasting month of Ramadan (the ninth month of the Islamic *Hijri* calendar), Muslims are required to abstain from eating, drinking, and having sexual intercourse from dawn (*suhoor*) until dusk (*iftaar*) (Jahrami et al., 2020). This Islamic ritual is one of the five tenets of the religion that all Muslims are obliged to observe if they are healthy (both physically and mentally) adults, not on a distant journey that is exhausting to the body, not pregnant or breastfeeding or menstruating (Faris et al., 2020; Trepanowski & Bloomer, 2010). Ramadan is a period devoted to prayers, feeding, alms‐giving, and other acts of good that Muslims adhere to, seeking proximity to God (Ghobary et al., 2013). This spiritual practice that accelerates faith has also been shown to have many positive benefits to mental and physical health and cognition (Faris et al., 2020; Gilavand & Fatahiasl, 2018; Khan et al., 2018). Many studies have explored associations between Ramadan intermittent fasting (RIF) and the various components of mental health, with the majority reporting an overall improvement (Briki et al., 2019; Molavi et al., 2016). For example, Ramadan fasting has been shown to significantly lower depression among people observing this ritual compared to other periods outside of the holy month, with no sex disparity (Erdem, 2018; Koushali et al., 2013; Maryam et al., 2010; Yousuf et al., 2021). A significant anxiety reduction has also been linked to RIF (Erdem, 2018; Maryam et al., 2010; Yousuf et al., 2021). In contrast, a study has reported a nonsignificant change for women compared to men (Mohammadi et al., 2001). An Iranian study conducted on university students during Ramadan (DR) found intrinsic religiosity to be associated with improved mental health aspects of the students, mitigating depression and anxiety and helping in coping with psychological distress when compared to before Ramadan (BR) (Gilavand & Fatahiasl, 2018).

The relationship between mental health, especially feeling anxious and depressed, physical health, and religiosity has been extensively explored across different populations and religions (Moreira‐Almeida et al., 2006; Moutinho et al., 2017; Ronneberg et al., 2016; Saunders et al., 2021). The evidence has led to suggest a possible role for religiosity within healthcare management (Hammad et al., 2022; Mishra et al., 2017). A 2019 meta‐analysis of Arab Muslims found a pooled mean correlation coefficient of –0.22 between religiosity and self‐reported anxiety symptoms (Abdel‐Khalek et al., 2019). Although this signifies negligible correlation, the analysis suggested that those with higher levels of religiosity may have enhanced coping strategies, which can help better manage anxiety‐related symptoms (Abdel‐Khalek et al., 2019). A cross‐sectional survey of 38,694 individuals from the French general population found that religious beliefs and observance were protective factors for self‐reported suicide attempts (Brito et al., 2021). However, the results are not homogenous, as religious beliefs were positively associated with psychotic symptoms/disorders, generalized anxiety disorder, and unipolar depression.

At a biological level, religiosity and spirituality appear to activate and possibly strengthen specific neurological pathways and regions (Rim et al., 2019). Evidence suggests that those who were religious/spiritual compared to their nonreligious/spiritual counterparts showed changes in their medial frontal cortex. Such changes have been previously linked to the maintenance of emotional states (Waugh et al., 2014); the orbitofrontal cortex, which has multiple functions and plays a crucial role in executive function and emotional states (Rudebeck & Rich, 2018); and the posterior cingulate cortex, which receives information from the orbitofrontal cortex (Rolls, 2019) and forms part of the limbic system, a network of interconnected regions, which is best known for the role it plays with an emotional response. Interestingly, telomere length is another biological finding positively related to religiosity (Shammas, 2011; Wang et al., 2020).

The evidence supporting the connection between religiosity and mental health is relatively consistent, regardless of age, religion, geographical location, and health status (Moreira‐Almeida et al., 2006; Moutinho et al., 2017; Ronneberg et al., 2016; Saunders et al., 2021; Weber & Pargament, 2014). While there is a developing research interest in the effects of Ramadan on Nigerian Muslims' perceptions of their lifestyle and health status (Sulaiman et al., 2022), there is a dearth of evidence linking religiosity in the context of Ramadan and mental health in this population. Multiple studies demonstrate a cross‐sectional link between religiosity and symptoms of anxiety and depression (Brito et al., 2021; Saunders et al., 2021; Shammas, 2011). Thus, in Muslim populations DR, where religiosity becomes all‐encompassing with additional religious practices, it is feasible to propose that mental health will improve in Ramadan when religiosity is likely to be more prominent. Therefore, we sought to examine potential alterations to some aspects of mental health, specifically anxiety and depression, amongst a large sample of Muslims BR and DR in adult Nigerians amidst the Coronavirus disease 2019 (COVID‐19) pandemic. To the best of the authors' knowledge, this is the first study that looked at the correlating factors of mental well‐being DR in Nigeria.

## 2 METHODS

### 2.1 Study design and respondents

A cross‐sectional web‐based survey was conducted from the May 9, 2021 (27th of Ramadan, 1442) to the June 4, 2021(29th of Shawwal, 1442) among Nigerian adults (at least 18 years old) who are Muslims residing in the country during the survey period and who fasted for 20 or more days during the month of Ramadan. We studied the mental well‐being of respondents over a period of 4 weeks BR and DR (between the April 12, 2021 and the May 12, 2021).

### 2.2 Sample size

We estimated a sample size of 687 respondents required for the study. If 687 respondents were recruited for the study, the study would have an effect size of 0.15, with a power of 0.99 and an alpha error of 0.1. We calculated using a bivariate correlation analysis model using two tail student *t*‐tests (Faul et al., 2007).

### 2.3 Study instrument and data collection

Data were collected with anonymity to exclude any identifying information using pretested, self‐administered, online questionnaires from previous studies (Arroll et al., 2010; Fisher & Ng, 2017; Plummer et al., 2016); while three other factors (intrinsic religiosity, spirituality, and eating habits) (Al Zaben et al., 2015; Athanasiadis et al., 2021; Fisher & Ng, 2017) were added to the questionnaire to suit the study objective. The questions were prepared on Google Forms (docs.google.com/forms) for the survey. The survey was conducted in English language as English is the main language in Nigeria. The survey took 20 min to complete and was conducted over 4 weeks to prevent any recall bias. The survey was collected through snowball and conveyance sampling. The survey questionnaire elicited information from the respondents about their sociodemographic characteristics,  eating habits (BR and DR) (Athanasiadis et al., 2021), mental well‐being (in terms of depression and anxiety) (Arroll et al., 2010; Plummer et al., 2016) BR and DR, spirituality (Fisher & Ng, 2017), and Muslim intrinsic religiosity (Al Zaben et al., 2015).

### 2.4 Sociodemographic characteristics

Survey respondents were asked about their age, sex, marital status, residence (rural or urban), educational status, occupational status (employed or unemployed), and total household income regarding the country's minimum household expenditure of NGN137,600 (USD 354) (Nigeria, n.d.) for families,  living status during the month of Ramadan (alone, with a friend, or with family), the number of days fasted DR.

### 2.5 Spirituality, intrinsic religiosity

Spirituality was assessed using the four‐item spiritual well‐being index (4‐ISWBI). The five options [very important (5), important (4), average (3), less important (2), not important (1)] in each question of the 4‐ISWBI were later collapsed into a Likert scale of three (very important, less important, not important) during analysis. The last three items of the 13‐item Muslim Religiosity Scale were adapted to assess for intrinsic religiosity of the respondents. These three questions (in my life, I experience the presence of Allah/God; my religious beliefs are what lie behind their whole approach to life; I try hard to carry religion over into all my other dealings in life) were scored on a five‐point Likert scale (definitely true, tends to be true, unsure, tends not to be true, definitely not true) and later collapsed into three (true, unsure, not true) during analysis.

### 2.6 Mental well‐being

Respondents' mental well‐being was assessed by using the adopted generalized anxiety disorder‐2 (GAD‐2) and Patient Health Questionnaire‐2 (PHQ‐2), respectively (Arroll et al., 2010; Plummer et al., 2016). For each question of the two scales, respondents were asked to score their responses based on a four‐point Likert scale [not at all (0), several days (1), more than half the days (2), nearly every day (3)], which was later collapsed into three (no change, less often, much often) during analysis.

### 2.7 Assessment of weight, height, and eating habits

Respondents were asked to self‐report their current body weights (in kilograms) and heights (in centimeters) which were used to calculate their corresponding body mass index (BMI) in kg/m^2^  during analysis, and classified into four categories: normal (18.5–24.9 kg/m^2^), underweight (<18.5 kg/m^2^), overweight (25.0–29.9 kg/m^2^) and obese (>30 kg/m^2^) (Weir & Jan, 2022). Respondents were assessed for eating habits by asking them about snacking, consuming large quantities of food and eating despite not feeling hungry BR and DR based on a seven‐point Likert scale [No (0), slightly less often (–1), slightly more often (+1), moderately less often (–2), moderately more often (+2), much less often (–3), much more often (+3)], which were later collapsed into three categories (no, less often, more often) during analysis.

### 2.8 Statistical analysis

Data collected were appropriately entered into a Microsoft Excel spreadsheet, cleaned and analyzed using STATA version 15.0 (StataCorp LLC, College Station, TX, USA). The mean and standard deviation (SD) were used to summarize continuous data. Categorical data were presented using frequencies and percentages. Respondents' psychological health BR and DR were compared using Pearson's chi‐square or Fisher's exact test as appropriate. Factors associated with symptoms of depression and anxiety were determined using multinomial regression analysis. Age, sex, and health state were considered as a priori confounding variables in all models. Independent variables with *p* < .10 at the bivariate level were included in the multivariate analysis. A backward stepwise regression was used, and the *p* values reported were from the likelihood ratio test. Adjusted odds ratios (aORs) and their 95% confidence intervals (CIs) were used to determine the strength and direction of the effect of factors associated with the dependent variables (depression and anxiety). Type I error was fixed at 5% for all tests.

## 3 RESULTS

### 3.1 Reliability of the study tools

Regarding reliability, the 4‐ISWBI and the intrinsic religiosity showed high internal consistency and high reliability, α = .86 and α = .75, respectively. Similarly, Cronbach's alpha of the adapted mental well‐being scale (GAD‐2 and PHQ‐2) was 0.84. Cronbach's alpha was also high, .84, for the total questionnaire, indicating that our instrument was robust and internally consistent.

### 3.2 Background characteristics of the respondents

A total of 770 respondents filled out the online questionnaires; however, 639 fulfilled the inclusion criteria and were included in the analysis. The majority (88.4%, *n* = 565) were between 21 and 40 years, with a mean age and SD of 28.5 ± 6.4 years. Almost two‐thirds (74.5%, *n* = 476) were men, and the vast majority, 71.7% (*n* = 458), lived in an urban area. About half were unemployed (48.4%, *n* = 309), and almost a quarter (23.5%, *n* = 150) were educated up to tertiary level. Three‐quarters (75.6%, *n* = 483) lived with their family. Full information on all demographic variables is presented in Table 1.

**TABLE 1:** Sociodemographic characteristics of the respondents *N* = 639

Variable *N* = 639	Frequency (*n*) (%)
**Age (in years)**	
≤20	48 (7.5)
21–40	565 (88.4)
41–60	26 (4.1)
**Sex**	
Female	163 (25.5)
**Marital status**	
Single	413 (64.6)
Married	217 (34.0)
Divorced	6 (0.9)
Widowed	3 (0.5)
**Residence**	
Rural	181 (28.3)
**Occupation**	
Employed	330 (51.6)
**Highest educational level**	
Tertiary or higher	150 (23.5)
Undergraduate	405 (63.3)
Secondary	81 (12.7)
Primary	3 (0.5)
**Household income relative to National Average**	
Top 20%	14 (2.2)
Upper 20%	101 15.8)
Middle 20%	354 (55.3)
Lower 20%	100 (15.7)
Lowest	70 (11.0)
**Living with during Ramadan**	
Alone	97 (15.2)
A friend	59 (9.2)
Family	483 (75.6)

### 3.3 Relationships and religiosity

Table 2 describes the spirituality (relationships) and intrinsic religiosity of the respondents. The majority of respondents considered spirituality to be very important in their life. A total of 85.1% (*n* = 543) agreed that a relationship with God was very important, and 83.9% (*n* = 536) considered their relationship with themselves very important. Relationships with other people were seen as very important by 74.1% (*n* = 480) of the respondents and the environment by 71.8% (*n* = 459). Almost all respondents (96.3%, *n* = 615) attested to the presence of God in their life (7.0%, *n* = 45) do not include religion in their life's dealings, and 1.5% (*n* = 10) were unsure about the role of religious beliefs in their approach to life.

**TABLE 2:** Spirituality (relationship) and intrinsic religiosity among the respondents, *N* = 639

Factor	Not important *n* (%)	Less important *n* (%)	Very important *n* (%)
Importance of relationship with self	48 (7.5)	55 (8.6)	536 (83.9)
Importance of relationship with God	47 (7.4)	48 (7.5)	544 (85.1)
Importance of relationship with other people	43 (6.7)	116 (18.2)	480 (75.1)
Importance of relationship with environment	43 (6.7)	137 (21.4)	459 (71.8)
**Factor**	**True *n* (%)**	**Not true *n* (%)**	**Unsure *n* (%)**
I experience the presence of God in my life	615 (96.3)	20 (3.1)	4 (0.6)
Religious beliefs are behind my whole approach to Life	594 (93.0)	35 (5.5)	10 (1.5)
I try hard to carry my Religion into all other life's dealings	577 (90.3)	45 (7.0)	17 (2.7)

### 3.4 Psychological well‐being before and during Ramadan

A higher proportion of respondents felt less depressed DR (56.5% vs. 61.3%).

Interest and pleasure in doing things were better DR than BR (*p*= 0.007) and respondents felt less nervous and anxious (60.7% vs. 57.1%) (Table 3).

**TABLE 3:** Respondents' mental well‐being before and during Ramadan, *N* = 639

**Factor**	**Before Ramadan**	**During Ramadan**	***p* Value**
**Little interest or pleasure in doing things that I enjoy**			
No change	151 (23.6)	173 (27.1)	**.007**
Less often	376 (58.9)	390 (61.1)	
More often	112(17.5)	76 (11.8)	
**Felt down, depressed or hopeless**			
No change	214 (33.5)	220 (34.4)	**<.001**
Less often	361 (56.5)	392 (61.3)	
More often	64 (10.0)	27 (4.3)	
**Felt nervous, anxious or on edge**			
No change	203 (31.8)	214 (33.5)	**<.001**
Less often	365 (57.1)	388 (60.7)	
More often	71 (11.1)	37 (5.8)	
**Unable to stop or control my worrying**			
No change	195 (30.5)	205 (32.1)	**<.001**
Less often	365 (57.1)	395 (61.8)	
More often	79 (12.4)	39 (6.1)	
**Consuming large quantities of food**			
Less often	374 (58.5)	392 (61.4)	.27
More often	113 (17.7)	91 (14.2)	
No	152 (23.8)	156 (24.4)	
**Eating despite not feeling hungry**			
Less often	351 (54.9)	378 (59.2)	**.001**
More often	108 (16.9)	72 (11.28)	
No	180 (28.2)	189 (29.6)	
**Snacking <0.001**			
Less often	410 (64.2)	453 (70.0)	
More often	131 (20.5)	56 (8.7)	
No	98 (15.3)	130 (20.3)	

### 3.5 Factors associated with the change in feeling depressed during Ramadan

Respondents' marital status, educational level, physical activity DR, and perceived importance of a relationship with self were independently associated with the feeling of depression DR. The odds of feeling more depressed DR were 60% lower in married respondents relative to their single counterparts (aOR: 0.4, 95% CI: 0.2–0.9). Similarly, respondents who were educated up to secondary school had fivefold increased odds of becoming more depressed DR (aOR: 4.7, 95% CI: 1.2–17.7) relative to those without any education. Furthermore, respondents who increased their physical activity DR were twice more likely to feel less depressed (aOR: 1.9, 95% CI: 1.5–2.5). Likewise, respondents who considered their relationship with self very important had a 70% reduction in odds of feeling more depressed (aOR: 0.3, 95% CI 0.1–0.7) (Table 4).

**TABLE 4:** Factors associated with the change in feeling depressed during Ramadan

**Covariate**	***Felt depressed less often***	***Felt depressed more often***	***p* Value**	***Felt depressed less often***	***Felt depressed more often***	***p* Value**
	**Crude OR (95% CI)**	**Crude OR (95% CI)**		**Adjusted OR (95% CI)** *	**Adjusted OR (95% CI)** *	
**Age (in years)**						
£20	Reference	Reference	.11	Reference	Reference	.62
20–40	1.7 (0.8–2.9)	0.5 (0.2–1.5)		1.6 (0.8–3.1)	0.9 (0.3–2.8)	
40–60	1.8 (0.4–4.2)	–		1.9 (0.6–6.0)	–	
**Marital status**						
Single	Reference	Reference	***.09***	Reference	Reference	***.02***
Married	0.9 (0.7–1.3)	0.3 (0.1–0.8)		1.0 (0.6–1.3)	0.4 (0.2–0.9)	
Divorced	4.1 (0.5–34.0)	–		4.4 (0.5–37.2)	–	
Widowed	1.2 (1.1–2.0)	–		1.1 (0.1–13.5)	–	
**Sex**						
Female	0.6 (0.4–1.0)	1.1 (0.6–2.2)	**.06**	0.7 (0.5–1.0)	1.0 (0.5–2.0)	**.07**
**Highest educational level**						
Tertiary or higher	Reference	Reference	***.04***	Reference	Reference	***.02***
Undergraduate	1.1 (0.7–1.9)	2.7 (0.9–6.9)		1.5 (1.0–2.3)	1.9 (0.5–5.9)	
Secondary	2.0 (1.0–3.0)	6.6 (1.9–23.0)		1.9 (1.0–3.5)	4.7 (1.2–17.7)	
Primary	0.6 (0.2–4.4)	–		0.3 (0.1–4.7)	–	
**Perceived health state during Ramadan**						
Good	Reference	Reference	.81	Reference		.66
Poor	1.3 (0.5–2.5)	0.5 (0.3–2.5)		1.1 (0.4–2.2)	0.6 (0.3–2.0)	
**Physical activity during Ramadan**						
Not changed	Reference	Reference	***.06***	Reference	Reference	***.04***
Increased	1.9 (1.3–2.6)	1.8 (0.9–3.6)		1.9 (1.5–2.5)	2.1 (0.9–4.4)	
Decreased	1.7 (1.1–2.6)	1.7 (0.8–3.7)		1.7 (1.0–2.6)	2.0 (0.8–4.9)	
**Relationship with self**						
Not important	Reference	Reference	***<.001***	Reference	Reference	***.01***
Less important	0.5 (0.2–1.3)	0.7 (0.5–2.6)		0.3 (0.1–1.3)	0.6 (0.5–2.5)	
Very important	0.6 (0.2–0.8)	0.3 (0.1–0.6)		0.8 (0.2–1.1)	0.3 (0.1–0.7)	
**Relationship with God**						
Not important	Reference		***<.001***	Reference		.35
Less important	0.5 (0.2–1.5)	–		0.7 (0.1–4.0)	–	
Very important	0.1 (0.1–0.6)	–		0.6 (0.2–3.2)	–	
**Relationship with other people**						
Not important	Reference	Reference	***<.002***	Reference	Reference	.55
Less important	0.6 (0.1–0.9)	1.4 (0.2–10.6)		0.3 (0.1–2.5)	1.2 (0.1–11.0)	
Very important	0.3 (0.2–0.6)	1.3 (0.2–10.7)		0.2 (0.1–2.0)	1.4 (0.2–11.7)	
**Relationship with environment**						
Not important	Reference	Reference	***.03***	Reference	Reference	.51
Less important	0.4 (0.3–1.0)	1.0 (0.2–4.5)		1.8 (0.5–6.3)	1.0 (0.2–4.5)	
Very important	0.4 (0.2–0.8)	0.5 (0.2–2.9)		2.3 (0.6–8.0)	0.7 (0.2–3.6)	

OR, odds ratio; CI, confidence interval; BMI, body mass index.

* Adjusted for age, sex, marital status, education, occupation, physical activity, perceived health state, consuming large quantities of food, eating despite not feeling hungry, relationship with self, relationship with God, relationship with other people, relationship with environment & Religious beliefs are behind the whole approach to life.

### 3.6 Factors associated with the change in the feeling of anxiety during Ramadan

Table 5 displays the factors associated with a change in anxiety DR. Educational level and physical activity DR were found to be independent risk factors for change in anxiety. Respondents that were educated up to secondary level were eightfold (aOR: 7.7, 95% CI: 3.2–27.5) more likely to be more anxious DR. On the other hand, respondents who decreased their physical activity were twice more likely to have increased anxiety (aOR: 1.6, 95% CI: 1.4–4.4).

**TABLE 5:** Factors associated with the change in anxiety during Ramadan

**Covariate**	***Felt anxious less often***	***Felt anxious more often***	***p* Value**	***Felt anxious less often***	***Felt anxious more often***	***p* Value**
	**Crude OR (95% CI)**	**Crude OR (95% CI)**		**Adjusted OR (95% CI)** *	**Adjusted OR (95% CI)** *	
**Age (years)**						
£20	Reference	Reference	***.06***	Reference	Reference	.29
20–40	2.4 (1.2–3.9)	0.9 (0.4–2.2)		1.9 (0.9–3.9)	1.4 (0.4–4.2)	
40–60	2.1 (0.8–5.9)	–		2.7 (0.9–8.6)	–	
**Sex**						
Female	0.8 (0.5–1.0)	1.1 (0.4–2.2)		0.7 (0.5–1.0)	1.1 (0.6–2.2)	
**Marital status**						
Single	Reference	Reference	***.04***	Reference	Reference	.31
Married	0.8 (0.6–1.4)	0.4 (0.2–0.7)		1.0 (0.7–1.4)	0.5 (0.2–1.4)	
Divorced	4.2 (0.4–31.4)	–		4.5 (0.5–39.7)	–	
Widowed	1.6 (0.4–10.7)	–		0.2 (0.1–3.7)	–	
**Highest educational status**						
Tertiary or higher	Reference	Reference	***.002***	Reference	Reference	***.01***
Undergraduate	1.5 (1.2–2.2)	3.8 (1.3–9.8)		1.5 (0.4–2.7)	3.3 (1.0–9.0)	
Secondary	1.7 (1.1–3.3)	9.0 (2.6–28.9)		1.7 (1.0–3.2)	7.7 (3.2–27.5)	
Primary	0.4 (0.4–5.6)	–		0.2 (0.1–3.6)	–	
**BMI**						
Normal	Reference	Reference	***.07***	Reference	Reference	.22
Underweight	1.0 (0.6–1.7)	0.6 (0.2–1.3)		1.3 (0.7–2.1)	0.7 (0.2–1.4)	
Overweight	0.9 (0.5–1.6)	0.4 (0.1–1.1)		1.0 (0.6–1.6)	0.4 (0.1–1.9)	
Obese	0.4 (0.2–1.3)	2.2 (0.9–3.9)		0.6 (0.3–1.6)	1.9 (0.9–4.1)	
**Physical activity during Ramadan**						
Not changed	Reference	Reference	***.003***	Reference	Reference	**.02**
Increased	1.7 (1.2–2.6)	2.8 (0.9–4.2)		1.8 (0.8–2.5)	1.6 (0.7–3.1)	
Decreased	1.7 (1.2–2.6)	2.0 (0.8–4.8)		1.6 (0.8–2.5)	1.6 (1.4–4.4)	
**Relationship with self**						
Not important	Reference	Reference	***<.001***	Reference		.07
Less important	0.6 (0.2–1.1)	1.8 (0.2–11.3)		0.5 (0.2–1.9)	1.9 (0.1–41.2)	
Very important	0.3 (0.2–0.6)	2.3 (1.5–11.4)		0.3 (0.1–1.1)	5.9 (0.4–10.4)	
**Relationship with God**						
Not important	Reference		***.02***	Reference		.24
Less important	0.7 (0.4–1.5)	0.5 (0.1–9.6)		1.4 (0.6–8.2)	0.9 (0.1–8.4)	
Very important	0.3 (0.1–0.6)	1.9 (0.2–12.7)		0.5 (0.1–2.6)	4.1 (0.1–15.0)	
**Relationship with other people**						
Not important	Reference		***.002***	Reference	Reference	.28
Less important	0.6 (0.2–0.8)	1.2 (0.2–11.0)		0.6 (0.1–3.3)	5.0 (0.2–13.7)	
Very important	0.5 (0.1–0.9)	1.4 (0.2–11.7)		0.4 (0.5–3.4)	6.5 (0.2–12.5)	
**Relationship with environment**						
Not important	Reference		*.03*	Reference		.29
Less important	0.4 (0.2–1.0)	–		0.8 (0.5–5.8)	–	
Very important	0.3 (0.2–0.8)	–		0.7 (0.5–8.0)	–	

OR, odds ratio; CI, confidence interval; BMI, body mass index.

* Adjusted for age, sex, marital status, occupation, marital status, perceived health state during Ramadan, relationship with self, relationship with God, relationship with the environment, and relationship with other people.

## 4 DISCUSSION

Our study found a significant improvement in mental well‐being among the respondents, and symptoms of depression and anxiety were found to be more prevalent BR. Interest and pleasure in doing things were also better during the month. Mental well‐being was found to be independently associated with sociodemographic characteristics, physical activity, and perceived relationships.

Our results suggest that RIF promotes psychological well‐being, which is consistent with several previous studies' results. A prospective study examining the effect of RIF on depression among people with type 2 diabetes mellitus found a significant reduction in depression among the respondents (Al‐Ozairi et al., 2019). Another research conducted among the Iranian population demonstrated that fasting has positive effects on psychological well‐being, with reduced depression and anxiety (Mousavi et al., 2014). Berthelot and colleagues conducted a meta‐analysis in 2021 that included 11 studies with a total of 1436 respondents examining the effectiveness of fasting interventions on mental health (Berthelot et al., 2021). The result showed that RIF has a positive effect on anxiety and depression. In addition to its positive effects on depression and anxiety, RIF has also been shown to have a diminishing impact on stress (Berthelot et al., 2021; Erdem, 2018; Koushali et al., 2013) and positive effects on mood and self‐esteem (Fond et al., 2013). These effects of RIF on mental well‐being have been attributed to the reductions in serum cortisol, and brain‐derived neurotrophic factor (BDNF) of fasting Muslims brought about by this Islamic ritual (Riat et al., 2021).

Many mechanisms can explain the therapeutic role of Ramadan on mental health, one of which is its lowering effect on body weight (for both obese and nonobese people) (Faris et al., 2020; Fernando et al., 2019; Jahrami et al., 2020). This is because obesity and being overweight have been positively correlated to increased incidence of both anxiety and depression (Fulton et al., 2022; Sharafi et al., 2020). This was earlier buttressed in a prospective cohort study of 25,180 men and women aged 19 to 55, which found anxiety and depression to be associated with increased body weight and increased incidence of obesity (Brumpton et al., 2013). Earlier than this, a 5‐year observational study of 2123 people aged 50 and older found obesity at baseline associated with an increased risk of depression 5 years later (Roberts et al., 2003). On the one hand, a reciprocal association between obesity/overweight with depression and/or anxiety has been reported (de Wit et al., 2010; Strine et al., 2008), while on the other hand, contrary findings were reported for depression (Roberts et al., 2003), and anxiety (Ejike, 2013). Furthermore, sex disparity has been observed in terms of obesity association with anxiety and depression. This was reported in an Australian study of 2.280 respondents aged 20–64 years, wherein obesity in women was found to be associated with greater anxiety and depression and less positive affect, but not in men (Jorm et al., 2003). Biological/molecular mechanisms have also been used to explain the beneficial effect of RIF on mental health. In a study from 2021, these therapeutic effects on mood‐related symptoms have been attributed to the reductions in serum cortisol, and BDNF of fasting Muslims brought about by RIF (Riat et al., 2021), although one study reported an increase in serotonin, BNDF and nerve growth factor in a cohort of “22 women” fasting DR (Bastani et al., 2017). Similarly, RIF has been found to lower the serum levels of proinflammatory cytokines Interleukin (IL), including IL‐1, IL‐6, and tumor necrosis factor α (Faris et al., 2019, 2012), which are elevated in the serum of depressed people (Young et al., 2014). These and other neurobiological factors have as well been reported to account for the observed effects of “diet restriction and intermittent fasting” even in non‐Ramadan fasting subjects (Fond et al., 2013; Igwe et al., 2021); there is yet to be any consensus to perfectly explain the precise nature of depression (Yang et al., 2020). Despite these vastly reported benefits of RIF on aspects of mental health, some studies have reported no or insignificant change (Hsouna et al., 2019; Nugraha et al., 2017). Cultural and lifestyle variations between populations could explain this disparity.

Furthermore, a large proportion of the respondents reported good mental health during the COVID‐19 lockdown. Nearly half of them agreed that the execution of their routine activities was good, despite the tremendous impact of the pandemic on global mental health (Rajkumar, 2020), which resulted in high levels of depression, stress and anxiety in different parts of the world (Ahmad et al., 2020; Shi et al., 2020; Wang et al., 2020a; Wang et al., 2020b), including Nigeria (Nri‐Ezedi et al., 2020; Olaseni et al., 2020). This could be explained by the role of religiousness and spirituality that characterized the study population (Carranza Esteban et al., 2021). This could further be explained by the Islamic faith and its role in alleviating the adverse effect of the pandemic and the Prophet Mohamed's (Peace be upon him)  teachings and directions regarding how to deal with pandemics (BinTaleb & Aseery, 2022).

In our study, the respondents reported changes to their eating behavior DR (Table 3). People usually eat two meals DR, one before dawn (Sahur) and another immediately after sunset (Iftar), but some individuals take an additional meal just before sleeping. There is a tendency to deliberately ingest extra amounts of food just before dawn to help endure the effects of hunger during the daytime (Kerimoglu et al., 2010). This is not just limited to food but also drinks, as fasting people drink nearly half more fluids than those not fasting within the fasting period (Kerimoglu et al., 2010). These habits (dietary restriction during the daytime and excessive eating at night) may seem to be a risk for the later development of eating disorders, which have been found to affect mental well‐being (Köster & Mojet, 2015). However, most studies examining the effects of RIF on disordered eating behavior showed no significant relationship between the two (Chia et al., 2018; Düzçeker et al., 2021; Erol et al., 2008).

This study found significant correlations between psychological well‐being and some sociodemographic characteristics (such as marital status and educational level), physical activity, religiosity, and spirituality. While all these were independently associated with the feeling of depression DR, only education and physical activity were found to have significant correlation with anxiety during the month. The relationship between depression and marital status in this study is highly significant, consistent with the results of several previous studies (Akhtar‐Danesh & Landeen, 2007; Grundström et al., 2021; Scarinci et al., 2002). For instance, a study examining the relationship between socioeconomic status indicators and depression among African women reported that women who have never married present with relatively higher levels of depression than those who were married (Scarinci et al., 2002). A similar result was found in another study conducted in the province of Ontario, Canada (Akhtar‐Danesh & Landeen, 2007). However, these contrast with an earlier similar study conducted in Northern Nigeria, where the author found that depression was diagnosed more frequently in married women than in single women (Ifabumuyi, 1983). Evidence highlights that the beneficial effect of marriage is mainly for men than women (Brown et al., 2000; Weissman et al., 1996).

It is generally believed that education has a protective effect on mental health. However, our result showed a positive correlation, as respondents who were educated up to secondary school were five and eight times more likely to be depressed and anxious, respectively. Conflicting results have been reported regarding the association between education and depression and anxiety, with many researchers reporting an inverse relationship (Andrews et al., 2001; Bjelland et al., 2008; Scarinci et al., 2002) and others proving the opposite (Akhtar‐Danesh & Landeen, 2007; Fryers et al., 2003). The protective effects of higher education on mental health vary across population subgroups. Generally, it has a more beneficial effect on women than men, whites than blacks, and people growing up in families with limited socioeconomic resources (Bjelland et al., 2008). Depression was noted to reduce with increasing education levels, particularly for highly educated people (Bjelland et al., 2008). However, anxiety is mainly associated with low education, and their relationship might be determined by genetic factors common to both (Tambs et al., 2012).

The multidimensional relationship between physical activity while fasting and mental health, in the context of COVID‐19, has been studied previously (Akbari et al., 2022; Ghram et al., 2021; Washif et al., 2022). The current research found an inverse relationship between physical activity and mental health status. Individuals that increased their physical activity during the period were noted to have reduced the likelihood of being depressed or hopeless by nearly half. This supports the findings of many previous studies (Legey et al., 2017; López‐Bueno et al., 2020; Raglin, 1990). A recent survey conducted among the Spanish adult population during the COVID‐19 lockdown in 2020 found that consistent physical activity was associated with a reduction in perceived anxiety and depression (López‐Bueno et al., 2020). Another study also obtained a negative correlation between physical activity and anxiety (Legey et al., 2017). Furthermore, regular moderate physical activity has been reported to protect against the development of depression (Raglin, 1990), and in fact, a sedentary lifestyle has been shown to be an essential risk factor for depression (Yates et al., 2020).

Another important finding of the current research was the negative correlation between perceived relationships, which define individuals' spirituality, and improved mental well‐being. It is worth mentioning that besides the physical aspect of RIF, the month provides an avenue for improving relationships as fasting individuals are encouraged to reassess and reestablish meaningful relationships with other people, the community, and the Creator. It teaches people to share the responsibilities of people, especially those less privileged, through showing mercy and humility and giving charity. These foster unity in society and strengthen interpersonal relationships, which could explain why most respondents in our study, 74.0% (*n* = 570), believe relationships are essential in their lives. These results are consistent with the findings of Kim et al., who highlighted that interpersonal relationships and other factors could lessen the symptoms of depression and anxiety (Kim et al., 2011). Surprisingly, we found no significant influence of intrinsic religiosity on depression or anxiety. This finding is indeed unexpected because religiosity has been described to have a profound impact on both physical health and psychological well‐being, and it contradicts the findings of some studies that found that intrinsic religiosity was strongly associated with fewer depressive symptoms and improved quality of life (Stroppa & Moreira‐Almeida, 2013) as well as with faster remission of depression (Koenig et al., 1998).

To the best of the authors' knowledge, this is the first study in Nigeria reviewing the impact of RIF on mental well‐being. We also correlated the impact of intrinsic religiosity and found it not to affect mental well‐being. The study is not without limitations.

Among the study's strengths is the relatively large sample size across the country. However, the respondents were not followed up before or after fasting since the study used a single‐point cross‐sectional design. A randomized controlled trial or a longitudinal study will shed more light on RIF effect on mental well‐being. As the study is a cross‐sectional study, it is difficult to establish causality in the relationships that we have identified. Furthermore, with the self‐reporting nature of the study, there is a risk of social‐desirability bias. Also, as the study involves both men and women, it is possible that some results will be affected by the monthly menstrual cycle. To minimize such bias, we asked the same set of questions BR and DR surveyed at a single point in time. We emphasize further prospective research with large samples in patients with established mental health and normal individuals to compare the impact of religiosity and spirituality and RIF.

## 5 CONCLUSION

This study found significant improvement in mental well‐being DR compared to the BR period despite the ongoing COVID‐ 19 pandemic. While marital status, education, physical activity, and spirituality were independently associated with depression DR, only education and physical activity were found to have significant associations with feelings of anxiety. All these factors were negatively correlated with both depression and anxiety, except education, which appeared to have a direct relationship with anxiety. Hence, the effect of RIF on mental well‐being needs further research with multicentric studies among different sets of ethnic populations. Such studies could lead to an understanding of the effect of RIF on mental health, which could have a significant impact on mental well‐being.

## AUTHOR CONTRIBUTIONS

MABK, SKS, and FIA  conceived and designed the study. SKS, FIA, MSM, YAK, FMD, MH, and SS  participated in the organization of the activities. SKS, FIA, MSM, YAK, FMD, MH, and SS  managed the activities and collected the data. FIA and MABK analyzed and interpreted the data. SKS, FIA, TA, MIF, SFH, and MABK drafted the article. SKS, SFJ, FIA, TA, MSM, YAK, FMD, AH, SS, MIF, SFH, and MABK revised the manuscript critically for important intellectual content. All authors read and approved the final article.

## FUNDING

This research did not receive any specific grant from funding agencies in the public, commercial, or not‐for‐profit sectors.

## CONFLICT OF INTEREST STATEMENT

The authors declare that there is no conflict of interest regarding the publication of this paper.

### PEER REVIEW

The peer review history for this article is available at https://publons.com/publon/10.1002/brb3.2990.

## Data Availability

The data that support the findings of this study are available from the corresponding author upon reasonable request.
